# The Nudity of the *Ego*. An Eckhartian Perspective on the Levinas/Derrida Debate on Alterity

**DOI:** 10.1080/00071773.2015.1100881

**Published:** 2016-02-05

**Authors:** Martina Roesner

**Affiliations:** ^a^ Institut für Bibelwissenschaft/Altes Testament, Katholisch-Theologische Fakultät, Universität Wien, Schenkenstr. 8–10, A-1010Wien, Austria

## Abstract

The present paper examines the Eckhartian motives in Derrida's critique of Levinas’ concept of the “Other”. The focus is put on the Husserlian concept of *alter ego* that is at the core of the debate between Levinas and Derrida. Against Levinas, Derrida argues that *alter* is not an epithet that expresses a mere accidental modification of the *ego*, but an indicator of radical exteriority. Interestingly enough, this position is virtually identical with Meister Eckhart's interpretation of the famous proposition from Exodus 3:14 “I am who I am”. Eckhart claims that the pronoun *ego* denotes the absolutely simple substance of the uncreated intellect, which can, by definition, never receive any accidental determination whatsoever. The reduplication of the “I am” is by no means tautological, but expresses the intra-divine dynamic of the Father who engenders the Son as his perfect equal and *alter ego*. This transcendental conception of egoity also governs the relationships between human beings: the ethical encounter with the “Other” requires that we consider them not primarily in their empirical, contingent existence but in the transcendental purity of their indeclinable *ego*, which is identical with the incessant act in which God knows himself in the Son as his absolutely Other. Thus, Meister Eckhart's approach proves, against Levinas, that it is possible to develop an “egological” philosophy that avoids the pitfalls of a naturalistic and potentially violent ontology of the subject.

## 1. Introduction

One of the most prominent features of twentieth-century philosophy is the profound crisis of the traditional paradigm of rationality that has been predominant in European culture since the beginnings of ancient Greek philosophy. The increasing awareness of the limits and shortcomings of the theoretical, foundational approach to reality has often been accompanied by a contraposition of Western philosophy to other, non-Occidental traditions of thought. The later Heidegger, for instance, refers to Japanese language and culture as a possible counter-paradigm to the Aristotelian, categorical model governing the relationship between thoughts, words and reality.^1^ 1 See Heidegger, *On the Way to Language*, especially pp. 42–54. His main charge against traditional metaphysics being that it subjects things to the grasp of utilitarian calculation, the reference to Asian culture is meant to inspire a different model of *Dasein*’s dwelling in a world conceived primarily not as a totality of objects but as primordial openness. For Levinas, by contrast, this Heideggerian project of a non-metaphysical way of thinking still remains trapped in the fundamental categories of Occidental philosophy, essentially because of its cosmological orientation. In his eyes, only the biblical, Jewish tradition offers a truly alternative form of rationality, because it privileges the ethical dimension of human existence over both the objectifying, theoretical knowledge of the world and the possible forms of a pre-rational “sojourn” (*Verweilen*) in it. More importantly, the reference to a different cultural paradigm does not have the same systematic significance for the two authors. Heidegger sees East Asian thought as one possibility among others to regain the pre-theoretic significance of things inside a non-technological world which has already, albeit briefly, been realized during the early, pre-Socratic period of Western philosophy.^2^ 2 See Heidegger, *On the Way to Language*, pp. 38–40, 46; Heidegger and Fink, *Heraclitus*, pp. 52–55. Levinas, in turn, considers the primacy of the ethical a specific trait of Judaism as opposed to Greek philosophy, thereby claiming for his own argumentative position a radical exteriority with regard to the tradition of thought he intends to criticize.^3^ 3 Of course, Levinas is not opposed to the Greek paradigm of philosophical rationality as such, but he nevertheless maintains the idea that the fundamentally ethical worldview, which he identifies with Judaism, enjoys superiority over rational consciousness and logic as it is represented by Greek philosophy. As Ephraim Meir puts it: “In *Ethics and Infinity*, Levinas maintained that the biblical and the philosophical thinking are not contradictory [ … ]. Yet, the God of the Bible remains for him in philosophy the criterion of the spirit. Levinas explicitly states that philosophy is not the place of the original significance of human existence. [ … ] Contrary to Hegel, Levinas was convinced that Judaism was not to be surpassed by philosophy. Jewish wisdom would even have advantages over the ‘Greek' one” (Meir, “Athens and Jerusalem in Levinas's *Difficult Freedom*”, pp. 5–6).


Although keenly aware of the justified claims of Levinas’ critique, Derrida employs a more nuanced approach to the problematic aspects of Occidental philosophy. To this effect, he refers to certain authors whose originality sets them apart from the mainstream of classical “onto-theology”, in particular to Meister Eckhart and Nicholas of Cusa. As a number of recent publications have shown, the influence of Meister Eckhart on Derrida's thought is by no means to be underestimated, especially with regard to the question of negative theology and the limits of language.^4^ 4 See Almond, “Negative Theology, Derrida, and the Critique of Presence”, pp. 150–165; Almond, “Doing Violence upon God”, pp. 325–347; Almond, “How *Not* to Deconstruct a Dominican”, 329–344; Newheiser, “Eckhart, Derrida, and the Gift of Love”. In this paper, we shall focus on the presence of Eckhartian motives in Derrida's critical rereading of the arguments Levinas puts forward against the supposed absence of true alterity in Husserl's phenomenological approach. The decision to engage Eckhart in a dialogue with twentieth-century phenomenology is by no means an arbitrary interpretative construction, but can be justified from a philosophical point of view. Contrary to a popular misconception, Eckhart's thought is in fact the exact opposite of irrational mysticism, for it contains an astonishing, innovative philosophy of the *ego* which, thanks to its proximity to the biblical tradition, remains unaffected by Levinas’ criticism of Greek philosophy.

As shall become clear in the course of the following pages, Derrida's defence of the Husserlian concept of *alter ego* follows closely the fundamental structure of Eckhart's transcendental egology, even without espousing all of its theological implications. Derrida obviously shares Eckhart's conviction that one can conceive the rational dignity of the human subject in terms of egoity without necessarily relapsing into a naturalistic or solipsistic paradigm of thought. His essay “Violence and Metaphysics” where this idea is developed contains only direct quotations of Eckhart's German sermons. It is therefore hard to determine if Derrida was equally familiar with the principal Latin writings, in which Eckhart exposes his conception of the *ego* from a more speculative viewpoint. However, our paper claims that Derrida's conception of the *ego* as it is developed in “Violence and Metaphysics” presents enough structural resemblances to Eckhart's theory of the intellectual “I” to make the hypothesis of a direct knowledge of his Latin writings at least reasonably plausible.

We shall start our analyses by recapitulating those aspects of Levinas’ phenomenology of alterity that seem particularly problematic or at least not entirely conclusive, then proceed to an exposition of Eckhart's philosophical conception of the *ego*, which appears as an *ante litteram* answer to Levinas’ criticism of traditional ontology, and conclude by highlighting the striking resemblances between the German Dominican's approach and the argumentation strategy that Derrida adopts in his defence of Husserl's phenomenology of intersubjectivity. Thus, Meister Eckhart assumes the position of an invisible hinge in the Derrida–Levinas debate on alterity: on the one hand, his specific metaphysical approach proves, against Levinas, that it is quite possible to conceive ethical intersubjectivity in terms of intellectual relationships between different *egos* without violently reducing the Other to a mere intentional object of thought; on the other hand, Eckhart's “egological” approach goes hand-in-hand with the development of a speculative grammar that anticipates Derrida's key idea, according to which difference and alterity never present themselves immediately but are necessarily mediated by a specific form of non-objectifying language.

## 2. The Problem of the *Alter Ego* as Keynote of Levinas’ Critique of Husserl's Phenomenology

Among all the works by Husserl published during his lifetime, probably none has raised more controversial reactions and received more divergent interpretations than the *Cartesian Meditations* (henceforward referred to as CM). Since first being issued in 1931 in a French translation by Levinas and Peiffer, this text has been considered the key reference to Husserl's phenomenology of intersubjectivity, especially as the greatest bulk of manuscripts dedicated to this topic did not become accessible until 1973 as vols. XIII–XV of the *Husserliana* edition. As much as the emphasis on the intersubjective constitution of the objective world in the CM represents a considerable advance with regard to the transcendental solitude of the *ego* in the *Ideas I*, this conception of intersubjectivity has nevertheless been criticized for the inconsistencies and inadequacies of the underlying phenomenological conception of the body. Although Husserl is at pains to stress the irreducible difference between the living organic body (*Leib*) and a mere physical thing (*Körper*), his approach in the CM seems to suggest that the constitution of human corporeity is still subordinate to the schema of theoretical intentionality. As a consequence, the animate, sensitive quality of the human body is not really presented as a phenomenal sphere *sui generis*, but appears more often than not as a mere addition to the primordial stratum (*Urschicht*) of physical, spatial extension.^5^ 5 See Husserl, *Cartesian Meditations*, pp. 95–100.


The critiques brought forward against the problematic aspects of Husserl's phenomenology of the body have taken in quite different directions. Merleau-Ponty, for instance, points out its shortcomings from a systematic point of view that, like the CM, still focuses on the phenomenological perspective of the individual. In his eyes, the relationship between each centre of transcendental consciousness and its own living body cannot be interpreted in terms of intentionality because it constitutes the essentially pre-conscious origin of any intentional relationship with noematic objects.^6^ 6 See Merleau-Ponty, *Phenomenology of Perception*, pp. 98–147. Thus, Merleau-Ponty does not so much criticize Husserl's approach as such, but rather its lack of phenomenological radicalism insofar as it still seeks to explain the specific status of the animate body as a corollary of the phenomenality of inanimate objects, instead of the reverse. Merleau-Ponty, however, leaves no doubt about the fact that his analyses still concern primarily the perceptions of the individual as such, i.e. with regard to the corporeal structure that is inalienably proper to it.^7^ 7 Ibid., pp. 369–409.


A far more severe and thoroughgoing criticism of Husserl's phenomenological approach in the CM has been formulated by Levinas. The insufficiency he discerns in Husserl's conception of intersubjectivity concerns just not the relationship between each individual *ego* and its own living body but, on the contrary, the ethical implications of any individualistic, *ego*-centred perspective in general. For Levinas, the very notion of *alter ego* bears witness to the more or less violent attempt to assimilate the otherness of the Other into the all-encompassing sphere of the primordial “I”. More precisely, the *alter* appears to him not so much as the indicator of true, independent exteriority but as a mere accidental modification of the absolute, indivisible substance of the *ego* in its undeclinable ipseity.^8^ 8 See Levinas, *Totality and Infinity*, p. 201. The key issue in this debate obviously concerns the underlying intention of Husserl's analyses. By linking the problem of the *alter ego* to the task of the constitution of a “world for all”, Husserl does not intend to reduce foreign subjectivity to a mere epiphenomenon of the sphere of natural objects but to inscribe it in a context of universal, rational validity that provides a reliable phenomenal fundament for responsible actions and, thereby, the constitution of an essentially ethical humanity.^9^ 9 See Husserl, *Cartesian Meditations*, pp. 131–136, 156. Levinas, however, takes this ideal of objectivity as an expression of ethical neutrality and accuses the concomitant “theoretical attitude” of reducing the metaphysical transcendence of the Other to a mere corollary of spatial exteriority in general.^10^ 10 See Levinas, *Time and the Other*, pp. 64–66.


In the CM, the Other's body is interpreted as the phenomenal place where the otherwise inaccessible sphere of a foreign subjectivity can be apperceived and thus indirectly experienced. Nevertheless, Husserl's principal concern is clearly not the perception of foreign corporeity as such, but its essential role for the establishment of a relationship between one's own *ego* and the *ego* of the Other as equally primordial origin of intentional acts of consciousness and rational actions. To put it differently, the other person's body is not considered with regard to its specific opacity, but rather in its relative transparency, which allows the psychic sphere of the *alter ego* to “shine through” and announce itself without ever becoming directly accessible to immediate, adequate intuition.^11^ 11 See Husserl, *Cartesian Meditations*, pp. 120–125. Of course, this “transparency” does not mean that the sphere of consciousness of the Other can be appropriated like an inner-worldly noematic object, but only that Husserl, unlike Levinas, still conceives this very inaccessibility of the Other's consciousness as an – albeit modified – form of intentional “experience”. See on this point Haworth, “Telepathy and Intersubjectivity in Derrida, Husserl and Levinas”, pp. 258–260. Thus, the phenomenological resistance and impenetrability of the body is apparently not a topic of reflection in its own right but still subordinate to the aims of a rationality that seeks to dominate its objects in the active medium of intelligible light. Husserl's conception of consciousness as an invisible but apperceptible centre of organization and orientation of a living being also accounts for the fact that he pays relatively little attention to the immanent differentiation of the body (i.e. head, hands, legs, feet, etc.), although the particular location of the body parts and their relationship to the organism as a whole play an essential role in the constitution of a specifically human world.^12^ 12 This lack of a detailed analysis of the human body structure and its immanent differentiation has justly been criticized by Hans Blumenberg (see Blumenberg, *Beschreibung des Menschen*, pp. 26–29, 37, 106–107, 144). Instead, the CM and other texts like *Thing and Space* tend to conceive the bodily structure as such in terms of potency (*ich kann*/“I can”), for it allows the subject to freely change its position with regard to any given spatial object.^13^ 13 See Husserl, *Cartesian Meditations*, pp. 118–131; Husserl, *Thing and Space*, pp. 73–88.


Levinas’ emphasis on the qualitative primacy of the “face” with regard to the rest of the body intends to remedy what he perceives as a residue of traditional ontology in Husserl's phenomenology of corporeity. The “face” is synonymous with the original, non-objectifiable presentation of an Otherness that transcends even the most radical differences expressible in categorical language, thereby putting an obstacle to any type of rationality conceived in terms of power. The “face” is not something that can be spoken *about* in the quasi-spatial distance of theoretical intentionality, but can only be directly addressed and spoken *to* as the manifestation of radical, non-logifiable difference. This fracture of the ontological categories is therefore not the result of an inner-logical, systematic consideration, but has its roots in a radically pre-objective affection of the subject that calls for an equally pre-objective linguistic paradigm. Levinas attributes the radical difference between the “face” and any natural object to its nudity and physical defencelessness, which is the ultimate reason for the ethical imperative “Thou shalt not commit murder”.^14^ 14 See Levinas, *Totality and Infinity*, p. 199. This conception of the Other consists mainly in positing a negative limit and barrier to the presumptions of potency displayed by traditional ontology, but it does not allow a positive overcoming of this philosophical paradigm from within its own conceptuality.

Despite the undeniably religious dimension of this “experience of infinity”, Levinas painstakingly emphasizes the distinction between his own biblically inspired notion of alterity and any form of mystical experience. Because the latter is traditionally defined as an a-verbal fusion with some “absolute” reality, it is the exact opposite of Levinas’ conception of ethical exteriority, which is essentially based on the idea of non-assimilation. In his eyes, mysticism amounts not only to the disappearance of true alterity, but also to an experience of plenitude that renders the language of solicitation superfluous.^15^ 15 See Levinas, *Difficult Freedom*, pp. 11–23, 100–102, 144–145; Levinas, “Is Ontology Fundamental?”, p. 8. According to Levinas, for the intersubjective relation to possess a genuinely ethical quality, it has to address the Other in the contingency and vulnerability proper to his bodily existence. That this is not the only way to avoid a monolithic conception of the *ego*, and that one can define mysticism otherwise than in terms of disappearance of all differences in pre-logical and pre-ethical darkness shall become clear in the course of our following analyses.^16^ 16 Levinas’ somewhat undifferentiated use of the concept of “mysticism” is critically discussed by Paul Rigby, “Levinas and Christian mysticism after Auschwitz”, pp. 309–334.


Aside from the ethical impact of its phenomenal appearance, the “face” implies another ontological rupture in that it is not “already given” in the synchronic medium of substantial presence. In *Time and the Other*, as well as in *Totality and Infinity*, Levinas refers to the father–son relationship as paradigmatic example of an experience of the radically Other above and beyond the ontological categories of identity and difference.^17^ 17 See Levinas, *Time and the Other*, pp. 90–94; Levinas, *Totality and Infinity*, pp. 267–269. His choice of this particular example is all but coincidental, for it implies that radical Otherness is not primarily experienced with regard to what already exists, but in relation to an original emergence to being. The people we encounter in our everyday-life are perceived as “Others” that are already part of our present horizon of experience. In the case of the father–son relationship, however, this phenomenon of exteriority is radicalized insofar as the engendering and birth of a previously non-existing child marks an additional de-possession of the subject with regard to the temporal horizon of its consciousness.^18^ 18 “In his early work Levinas presents eroticism as the hidden heart of social life. From here it is a small step to the phenomenon of fertility. [ … ] From now on, being and time mean: being and fertility. After all, it is fertility which forms the bridge between the time of the self and the time of the other. I can experience the time which starts with my child's life as being my own and at the same time not being my own” (van Riessen, *Man as Place of God*, p. 32). If true intersubjectivity only occurred between already existing persons, we could give in to the reassuring illusion that in virtue of this synchronicity, at least the transcendental sphere of the ethical encounter with the heterogeneous Other remains closed and homogeneous. From this perspective, the relationship between father and son is paramount insofar as it shifts the traditional philosophical understanding of “immortality” and “infinity”, conceived as essential properties of the singular (transcendental) subject, to the complex, diachronic structure of generative intersubjectivity. As paradoxical as it might seem, the Levinasian subject partakes of immortality to the precise extent that it ceases to insist on its own self-preservation and permits itself to be drawn outside the boundaries of its own selfness. Although the son owes his very existence to his father, the possibility of the younger generation to outlive their elders marks a discontinuity between them, which can no longer be overcome from the viewpoint of theoretical constitution.^19^ 19 See Levinas, *Totality and Infinity*, pp. 269, 271–272. While the son cannot come to exist without his father, he incarnates a temporal intentionality towards the future that radically transcends the individual self of his progenitor.

Even without explicitly putting it in these terms, Levinas’ emphasis on “fertility” as paradigmatic experience of radical transcendence seems to insinuate that Husserl's effort to explain the phenomenality of the *alter ego* from within the immanent sphere of transcendental consciousness is no more than the unsuccessful and, therefore, barren attempt of the *ego* to create alterity by engaging in a narcissistic play with its own reflection.^20^ 20 See Levinas, *Totality and Infinity*, p. 277. To Levinas, the fact that Husserl still speaks of the “constitution” of the *alter ego* and apparently subordinates it to the constitution of a world of material objects seems sufficient evidence to conclude that Husserl still considers the relationship with the Other as the unfolding and the actualization of a potency already present in the subject itself, rather than an experience of truly heterogeneous exteriority that disrupts the immanent consistency of the monadic *ego*.^21^ 21 Of course, one could object that Levinas does not want to eliminate the Husserlian *ego* altogether, but rather have it constituted by the “call” of the Other. The question is, however, if we have to conceive the ethical relationship between *ego* and its human Other in terms of metaphysical “primacy” at all or if both are always already correlated to one another in a dynamic which is the intra-human translation of their eternal generative relationship with the divine Other. As we shall see, Meister Eckhart's approach to ethical intersubjectivity is based on this second model. Despite his efforts to analyze the phenomenological specificity of the experience of a foreign subjectivity, as well as its profoundly ethical implications, Husserl therefore still talks *about* the *alter ego* according to the classical form of philosophy as theoretical *epistêmê*.^22^ 22 See Levinas, *Totality and Infinity*, p. 210. Hence Levinas’ claim that the blind spots of Occidental philosophy cannot be cured from within the Greek paradigm of thought – to which he is convinced Husserl still belongs – but only from the viewpoint of the Jewish-biblical tradition that envisages the Other already in terms of ethical concern and not indirectly via the cognitive relationship with inner-worldly objects.^23^ 23 Ibid., pp. 198–201.


From this perspective, Levinas’ paradigm of paternity and sonship has undoubtedly the merit of emphasizing the creative character of intersubjective relationships, but its argumentative power is somewhat diminished by the fact that it appears as a contingent counter-example taken from the factual world and, therefore, devoid of any transcendental necessity whatsoever. Levinas does mention the fact that the father–son relationship exceeds its merely naturalistic significance,^24^ 24 See Levinas, *Time and the Other*, p. 92; Levinas, *Totality and Infinity*, pp. 272–273. but he does not really explore the possibility of thinking the “fertility” of intersubjective relationships in terms of non-biological generation so as to make it applicable to human beings in general, regardless of their familial status. This is a point we shall return to later when dealing with Meister Eckhart's philosophical approach.

Legitimate as his intentions may be with regard to the fundamental ethical dimension of human existence, Levinas’ argumentation seems questionable on at least three points. First, he presents Husserl's conception of the constitutional relationship between *ego* and *alter ego* inside the framework of an objective “world for all” and his own counter-paradigm of ethical solicitation by the Other as mutually exclusive, at least as far as the supposed constitutive “primacy” of one's own *ego* over the Other's *ego* is concerned.^25^ 25 Despite his repeated claims that he still considers himself deeply indebted to Husserl's phenomenological project, Levinas in fact sharply criticizes its very foundation, i.e. the dominance of the cognitive dimension in our relationship with the Other (see on this point William Large, “On the meaning of the word ‘other' in Levinas”, pp. 37–38, 43–45). One could ask, however, whether every “cognitive” or “theoretical” attitude is necessarily synonymous with considering the Other as a mere intentional “object” of thought or whether there are other forms of intellectual relationality which do not absorb but, on the contrary, constitute the idea of ethical exteriority. This aspect is highly problematic in that the overemphasis on the “radically Other” seems to entail a complete equivocity between the language of ethics and the language of objective, theoretic knowledge of the world, as if the latter was automatically and necessarily synonymous of a “fallen”, inauthentic, and inherently violent form of rationality. Second, Levinas limits himself to contrasting the classical conception of transcendental subjectivity with those contingent aspects of human existence that exceed the realm of traditional philosophical conceptuality and transcendental consciousness (e.g. physical vulnerability, fatigue, insomnia, erotic attraction, etc.). In doing so, however, he tacitly presupposes without any further proof that the unilateral aspects of traditional ontology cannot be corrected and overcome from within the sphere of philosophical thought itself but only from an external point of view. Third, Levinas apparently takes for granted that Occidental philosophy is a more or less homogeneous totality, dominated from beginning to end by the objectifying, naturalistic categories established by Greek philosophy. This presupposition too is questionable, because it repeats the anti-ethical gesture of assimilation, justly criticized by Levinas in the context of intersubjective relationships, with regard to the heterogeneous phenomenality of philosophy itself.

As shall become evident, none of these three assumptions is stringent, and an unbiased study of the question may well reveal that Levinas’ tendency to postulate a profound difference, if not opposition, between the Greek and the Jewish-biblical paradigms of thought still remains tributary to the same logic he intends to denounce. Before turning to Derrida's critical rereading of Levinas’ theses, we will give a closer look at those aspects of Meister Eckhart's philosophical and theological approach that will prove to be of particular relevance to Derrida. In the context of our present discussion, Eckhart's thought is of special importance for at least three reasons: first, he offers an interpretation of the relationship *ego/alter ego* that exceeds the ontological, finite paradigm of identity and difference criticized by Levinas and puts particular emphasis on the essentially dynamic, relational, and generative character of the *ego*.^26^ 26 Certain similarities between Eckhart's and Levinas’ thought have been highlighted by Oliver Davies (see Davies, “Beyond the Language of Being”, pp. 32–40). His focus, however, lies more on their common criticism of the language of traditional onto-theology than on the question as to whether Meister Eckhart's approach can serve as a counter-example to Levinas’ global interpretation of Occidental philosophy. Second, Eckhart's thought is at least as profoundly marked by the biblical tradition and the exegetical approach of the Jewish thinker Maimonides as by pagan Greek and Latin authors. His approach proves that well before Levinas, certain Occidental philosophers and theologians have already recognized the ontological paradigm of natural objects as inapplicable to subjectivity in general and the relationship to the Other in particular. Instead of opposing the “pagan” and the “biblical” traditions of thought, Eckhart argues, however, that the One who manifests himself by saying “I am who I am” *is* the very essence of philosophical *noûs* in its transcendental nudity and radical exposure, i.e. divested of its garb of naturalistic properties. And third, Eckhart's speculative interpretation of the relationship between *ego* and *alter ego* is accompanied by the development of a transcendental syntax and grammar that pre-figures Derrida's analysis of the Husserlian notion of *alter ego* as expression of true exteriority and non-accidental difference.

## 3. Meister Eckhart's “Egology of Exodus”

Although Meister Eckhart is often considered one of the most famous mystics of the Middle Ages, there is hardly a medieval thinker to whom Levinas’ criticism of the fusional and potentially unethical orientation of mystical religiosity is less applicable.^27^ 27 See Rigby, “Levinas and Christian mysticism after Auschwitz”, pp. 325–326. On the contrary, Eckhart's sermons contain a surprisingly harsh criticism of the numerous contemporary forms of “experimental mysticism”, which give particular importance to ecstasies, raptures, visions and other supranatural phenomena that are supposed to accompany the mystical union of the soul with God. A fine psychologist as well as a first-class philosopher and theologian, Eckhart is well aware of the fact that these forms of “religious experiences”, while not necessarily delusional or heretic in nature, may lead the believer to be so completely wrapped up in their individual interiority and their “personal spirituality” that they neglect the most elementary forms of ethical behaviour and Christian charity towards their neighbour.^28^ 28 See Meister Eckhart, *Predigt* (= “sermon”, henceforward quoted as *Pr*.) 86, in *Werke*, vol. II, p. 211; for the English text, see *The Complete Mystical Works of Meister Eckhart*, p. 84. This does not mean that for Eckhart, there is no union between God and the soul. However, because this union is located in the intellectual “ground” of the soul, it is precisely not “experienced” through the ecstatic disappearance of rational consciousness, but on the contrary, manifests itself in the spontaneous effectivity (*Wirken*) and ethical fruitfulness of the soul in the external world.^29^ 29 See Meister Eckhart, *Reden der Unterweisung* (= “The Talks of Instruction”), ch. 10, W II, p. 363; for the English text, see *The Complete Mystical Works of Meister Eckhart*, pp. 496–497. All the believer's claims of being “one with God” are null and void if their acts towards their neighbour do not translate the essence of uncreated justice, which Eckhart places even above God as “object” of religious faith.^30^ 30 See Meister Eckhart, *Pr*. 6, W I, pp. 79–81; for the English text, see *The Complete Mystical Works of Meister Eckhart*, pp. 329–331. This position reveals an astonishing proximity to Levinas’ emphasis on the absolute primacy of the ethical with regard to both theoretical knowledge of the world and religious practice. Eckhart would have wholeheartedly agreed with Levinas that many so-called “mystical experiences” are likely to be no more than expressions of pseudo-religious self-absorption in disguise.

Unlike Levinas’ approach, however, Eckhart's profoundly ethical conception of Christianity does not stem from a simple rejection of traditional mysticism or theoretical philosophy, but is rooted in his particular metaphysical conception of the intellect, and especially the *ego*. At first sight, the highly speculative orientation of Eckhart's thought seems rather surprising, because for the most part his Scholastic opus comprises exegetical commentaries on different books of the Bible. A closer look reveals, however, that the contents belie the literary form, for Eckhart is clearly much less concerned with the historical sense of the biblical text than with its systematic interpretation in accordance with certain philosophical principles.^31^ 31 See Meister Eckhart, *Expositio sancti evangelii secundum Iohannem* (henceforward quoted as *In Ioh.*) no. 2, in *Die lateinischen Werke* ( = LW) III, p. 4; for the English translation, see *Meister Eckhart. The Essential Sermons, Commentaries, Treatises, and Defense*, pp. 122–123. This rather audacious theological endeavour goes far beyond the attempts made by Thomas Aquinas and other scholastic thinkers to prove the dignity of revealed theology as “science” in the Aristotelian sense. To them, even if Christian *sacra doctrina* can be interpreted according to the methodological criteria of scientific *epistêmê*, there is still an inextinguishable difference between Scripture-based theology on the one hand and metaphysics, or philosophical theology, on the other. Eckhart claims, by contrast, that Holy Writ and Aristotelian first philosophy teach exactly the same contents, albeit in different ways,^32^ 32 See Meister Eckhart, *In Ioh.* no. 185, LW III, pp. 154–155 (no English translation available). and makes no secret of the fact that he owes the idea of a perfect coincidence between the Bible and Aristotelian philosophy to the Jewish thinker Maimonides.^33^ 33 See Meister Eckhart, *Liber parabolarum Genesis* (henceforward quoted as *In Gen.* II) nos. 1–7, LW I, pp. 447–456; for the English translation, see *Meister Eckhart. The Essential Sermons, Commentaries, Treatises, and Defense*, pp. 92–95. See also Schwartz, “Meister Eckharts Schriftauslegung als maimonidisches Projekt”, pp. 173–208. He adopts the latter's fundamental thesis, according to which the Bible provides not only ethical and spiritual guidance but can and must also be read as a treatise on natural philosophy and metaphysics. The Maimonidean influence on Eckhart's thought is particularly visible in his interpretation of those passages of the Bible that deal with God's self-revelation and the related problem of the adequate or inadequate rendering of the divine names in human language.

Despite the conspicuous presence of Aristotelian concepts and topics throughout his works, the German Dominican does not adopt the peripatetic notion of substance as the ultimate fundament of his own metaphysics. More precisely speaking, Eckhart radicalizes the Aristotelian definition of *ousia*, which is essentially inspired by the paradigm of natural objects tainted with divisibility and contingent existence. In Aristotelian terms, the unity of natural substances is only a relative one, because it still allows of a multiplicity of accidental properties. What is even more important is that their unity and identity is something that can be recognized and predicated upon *from outside*, but not something the substance itself could be consciously aware of. Hence, the unity of the *ousia* is, properly speaking, a function of the intellect, which consists in singling out certain elements from the perpetual fluctuation of the phenomenal world and considering them as accidental properties of a certain substrate.

The predicative complexity of the Aristotelian system of categories leads Eckhart to the conclusion that the intellectual *ego*, which knows and understands nature according to these fundamental concepts, must be “pure substance” (*mera substantia*), i.e. substance in an absolute, original sense.^34^ 34 See Meister Eckhart, *Expositio libri Exodi* (henceforward quoted as *In Exod*.) nos. 14–16, LW II, pp. 20–22 (no English translation available); Meister Eckhart, *Sermones et Lectiones super Ecclesiastici* (henceforward referred to as *Super Eccl.*) no. 10, LW II, pp. 239–240 (no English translation available). Because multiplicity presupposes simplicity, the relative unity of natural substances is surpassed by, and dependent on, the indivisible unity of the intellect that knows them in the light of their universal, uncreated form. The difference between, on the one hand, the mode of being of the intellect, and on the other hand, the sphere of natural things is not only a question of degree, but implies an ontological quantum leap. Eckhart does not consider the intellect as *one* (albeit the highest) faculty of the human soul among others, but locates it in the same sphere of uncreatedness as the divine intellect itself. This particular conception of the relationship between the intelligible sphere and the sphere of natural beings is mirrored in the ontological hierarchy Eckhart establishes between the different transcendental properties. While Thomas Aquinas and most other Scholastic thinkers teach the horizontal convertibility of *ens*, *unum*, *verum* and *bonum*,^35^ 35 See Thomas Aquinas, *Summa Theologiae* I, q. 5, a. 1–3; q. 16, a. 3–4. For a more detailed discussion of this topic, see Aertsen, *Medieval Philosophy and the Transcendentals*. Eckhart reserves the *unum* and the *verum* for the sphere of uncreated reality and relegates the *ens* and the *bonum* to the level of created beings.^36^ 36 See Meister Eckhart, *Expositio libri Genesis* (henceforward quoted as *In Gen.* I) no. 68, LW I, p. 232 (no English translation available); Meister Eckhart, *Quaestio Parisiensis* I, nos. 4–8, LW V, pp. 40–45 (no English translation available).


This vertical hierarchization of the transcendentals entails a restriction with regard to the applicability of other philosophical principles, especially the four Aristotelian causes. Because the sphere of the *verum* is not subject to change, it is beyond the realm of efficient and final causality, both of which are proper to natural beings. Only form and (intelligible) matter apply to the sphere of the intellect, which means that its dynamism cannot be conceived in terms of exterior, physical causality.^37^ 37 See Meister Eckhart, *In Ioh*. no. 336, LW III, p. 284 (no English translation available). Aristotle had already recognized this distinction by emphasizing that the “good” does not apply to purely intelligible objects like mathematical entities, but only to real, extra-mental objects.^38^ 38 See Aristotle, *Metaphysics* III 2, 996 a 29–32. As a consequence, his conception of the divine *noêsis noêseôs* equates the idea of pure intelligibility with that of complete immobility and autarchic self-relatedness. The first principle of the cosmos is the supreme good only insofar as it is the ultimate goal all other beings tend to, but it is not overflowing goodness that generously communicates its own internal richness and intellectual bliss to the rest of reality.^39^ 39 See Aristotle, *Metaphysics* XII 7, 1072 b 1–30.


To be true, the Aristotelian notion of the divine can be seen as a paradigmatic example of the arrogant attitude of cognitive self-absorption and unethical self-centredness Levinas sees at work in virtually all the Occidental philosophers. Eckhart's philosophical and theological project, however, manages to escape this criticism. His approach is unique in that it blends the Aristotelian theory of the separate divine *noûs* with the biblical notion of God the Creator who not only manifests himself through his works, but also engages in a direct dialogue with humankind and invites human beings to pursue this dialogue in their mutual ethical relationships. To be precise, these two aspects are one and the same inasmuch as the creation of the sensible, material world is nothing else but the result of the groundless dynamic of the divine origin that recognizes and pronounces itself in the Word.^40^ 40 See Meister Eckhart, *In Ioh*. no. 73, LW III, p. 61; for the English translation see *Meister Eckhart. The Essential Sermons, Commentaries, Treatises, and Defense*, p. 148. Even with regard to the world of material things, God's creative action does not consist in the fabrication of exterior objects subsisting independently of their origin. Rather, what appears to us as the autonomous, extra-divine sphere of nature is part and parcel of the overflowing dynamic in which God engenders his Other from all eternity. Without the generation of the Son, i.e. the Word, there simply would be no such thing as a natural world. What distinguishes nature from God himself is therefore no more than a particular perspective, i.e. the possibility of considering the world *as if it were not* the result of the one and indivisible divine self-generation.^41^ 41 “Nota quod omnis creatura duplex habet esse. Unum in causis suis originalibus, saltem in verbo dei; et hoc est esse firmum et stabile [ … ]. Aliud est esse rerum extra in rerum natura, quod habent res in forma propria. Primum est esse virtuale, secundum est esse formale, quod plerumque infirmum et variabile”. – “Notice that every creature has a twofold being. One is in its original causes, or at least in God's word, and this is a solid and stable being. [ … ]. The other one is the being things have in the external reality, according to their proper form. The first is virtual being, the second is formal being, which, in most cases, is weak and changeable” (Meister Eckhart, *In Gen.* I no. 77, LW I, p. 238; the translation is ours).


Although firmly centred on the idea that God is intellect in its highest and purest form and that man's beatitude consists likewise in a specific form of intellectual knowledge, Eckhart's metaphysics manages to avoid the pitfalls of traditional Greek ontology by conceiving rational subjectivity according to a completely different paradigm than the world of natural objects. God's overflowing, creative potency is, in fact, not some anonymous principle we only can infer *a posteriori* from the structure of the created world; rather, it is intellectual selfness in its utmost purity, which addresses itself to the human intellect in order to be recognized as such. With regard to the intellect, therefore, “relation” is neither the weakest nor the least important of all categories, but on the contrary, the *only* category applicable to the pure substance of the *ego*, in both its divine and its human expression.^42^ 42 See Meister Eckhart, *In Exod*. nos. 63–65, LW II, pp. 67–70 (no English translation available).


The core passage for Eckhart's divine “egology” is his commentary on the famous verse in Exodus 3:14, where Yahweh manifests himself to Moses by saying “I am who I am”. Eckhart analyzes the corresponding Latin sentence *Ego sum qui sum* by examining first, the meaning of each word separately and then, the structure of the sentence as a whole. Above and beyond the objectives of traditional biblical hermeneutics, Eckhart's interpretation of the aforementioned verse has a highly philosophical thrust, for it aims to develop a transcendental grammar that exceeds the classical subject–predicate logic of Aristotelian metaphysics. Concerning the first term of this sentence, Eckhart claims that the pronoun *ego* does not merely replace the subject (i.e. the underlying “substrate” of the sentence), but manifests the *energeia* of the one who speaks. The purely deictic nature of this expression does not indicate a lack of predicative determination but rather its excess, because the self-consciousness of the *ego* is the transcendental condition for any subsequent affirmation or negation concerning objective states-of-things. The performative purity of the *ego* conceived as act, however, does not admit of any determination by “this or that” accidental property.^43^ 43 See Meister Eckhart, *In Exod.* no. 14, LW II, p. 20 (no English translation available). Its utter simplicity is without any determinate quality, but this does not mean that it merges into the neutral sphere of a-personal anonymity. On the contrary: *ego* stands for the highest principle of subjective identity insofar as it marks the breakthrough-point of intellectual spontaneity and liberty that can neither be deduced from, nor reduced to, natural causes and determinations.^44^ 44 See Meister Eckhart, *Super Eccl.* no. 8, LW II, pp. 235–236 (no English translation available).


It is important to notice that there is a marked difference between Eckhart's conception of the *ego* and the modern Cartesian philosophy of subjectivity. Whereas the latter considers the human *ego* from a minimalistic viewpoint as the ultimate residue of methodical scepticism, the German Dominican does not admit of any true egoity beside God himself. His *ego* is the plenitude of reality at its highest, whose absolute singularity is rich enough to forgo the mechanisms of monolithic self-conservation. In fact, God does not limit himself to saying “I” or at most “I am”, but pronounces the phrase “I am – who I am” that opens up the space of true intersubjective encounter. For Eckhart, the formulation *Ego sum qui sum* is not a mere tautology, but the expression of the divine self-engendering, where the monadic origin (i.e. the first *sum*) reflects its own loving ardour in an instance (the second *sum*) that partakes in the same abysmal spontaneity and liberty as its progenitor.^45^ 45 See Meister Eckhart, *In Exod.* no. 16, LW II, pp. 21–22. Between the origin and its offspring, there is no ontological difference allowing of a subject–predicate relationship, but only the distance necessary for two different configurations of the same absoluteness to recognize each other as such. The *alter ego* engendered by the divine *ego* is therefore not a mere accidental addition or modification but a radical, co-original form of alterity that allows the being-origin of its origin to become manifest.^46^ 46 “[ … ] nec est nec intelligitur pater sine filio et e converso, et per consequens filius non excludit nec tacet, sed enarrat patrem esse patrem”. – “The Father neither exists nor is known without the Son and vice versa, and consequently, the Son neither excludes nor conceals but openly tells the Father's being-a-Father” (Meister Eckhart, *In Ioh.* no. 197, LW III, p. 166; the translation is ours). Eckhart's Trinitarian interpretation of Exodus 3:14 therefore implies a profound reversal of the classical ontological hierarchy between cause and effect: the Father could neither be, nor become known, without the Son, for the Son is the cause of the Father's being-a-Father and without him, the Father could not even manifest his potency.^47^ 47 Interestingly enough, in *Totality and Infinity*, p. 277 Levinas uses an almost identical expression with regard to human paternity and sonship, but he does not seem to have noticed that this very schema of primordial correlation and mutual “causation” has Trinitarian origins.


As far as the intra-divine life is concerned, Eckhart's interpretation of the Father–Son relationship in terms of reflective intellectual self-recognition is, for the most part, in conformity with contemporary Scholastic teaching.^48^ 48 “Sic igitur processio verbi in divinis habet rationem generationis. Procedit enim per modum intelligibilis actionis, quae est operatio vitae, et a principio coniuncto, ut supra iam dictum est, et secundum rationem similitudinis, quia conceptio intellectus est similitudo rei intellectae”. – “So in this manner the procession of the Word in God is generation; for He proceeds by way of intelligible action, which is a vital operation, from a conjoined principle (as above described): by way of similitude, inasmuch as the concept of the intellect is a likeness of the object conceived” (Thomas Aquinas, *Summa theologiae* I, q. 27, a. 2 c; for the English text, see *The Summa Theologica*); see also Bonaventure, *Quaestiones disputatae de mysterio Trinitatis*, q. 5, a. 1, ad 8; for the English text, see *Disputed Questions on the Mystery of the Trinity*; Bonaventure, *De reductione artium ad theologiam,* no. 16; for the English text, see *On the Reduction of the Arts to Theology*. It is his univocal transposition of the logic of divine self-engendering to the sphere of human subjectivity and intersubjectivity that veers away from traditional theology and philosophy. Because for Eckhart, there is no true self besides the divine *ego*, the relationships between human persons too have to obey the Trinitarian schema of absolutely original, non-contingent intersubjectivity. As long as I consider the other person according to their qualities, properties, abilities, bodily appearance, etc., I subject them to the Aristotelian logic of substance and accidents. Only if I put into brackets “this or that” aspect of the Other and consider them as *ego*, that is, in the transcendental purity of their being-a-groundless-origin, I see them from the viewpoint of true, disinterested love.^49^ 49 See Meister Eckhart, *Super Eccl.* no. 9, LW II, p. 238 (no English translation available). There is, therefore, no contradiction whatsoever between the perspective of intellectual knowledge and that of absolute love, for both consist in viewing the Other as they are in themselves, apart from “this or that” property that belongs to the dimension of their created, natural being. Like Levinas, Meister Eckhart considers the relationship of the *ego* with its Other not as a mere corollary of the relationship with inner-worldly objects, but on the contrary, as the primordial origin and transcendental ground for any experience of the natural world.^50^ 50 See Meister Eckhart, *In Exod.* no. 10, LW II, p. 240 (no English translation available). Unlike Levinas, however, Eckhart does not consider the profoundly ethical dimension of this relationship and its essentially intellectual character as mutually exclusive, quite the contrary: even if the Other did not have a “face” but manifested themselves to us in other ways, we would still owe them justice by virtue of their uncreated, naked “I”.^51^ 51 See Meister Eckhart, *Pr.* 28, W I, pp. 321–325; for the English text, see *The Complete Mystical Works of Meister Eckhart*, pp. 131–132.


In Eckhart's transcendental grammar of intersubjectivity, the structure of the sentence “I am who I am” does not mirror the relationship between the substrate of predication and its more or less numerous accidents but reflects the space of verbal manifestation between two egoic instances that are one in their common incapacity of inhering in whatever or whomever else. In the relationship between the *ego* and the *alter ego*, the *alter* is therefore no accidental addition to the self-same subject, but marks the space of primordial exteriority, in which egoity accedes to itself *as* groundless, non-objectifying distance and otherness. Hence, Eckhart's ethics, unlike Levinas’, is not based on the notion of overwhelming, immediate presentation, but considers the Other from the viewpoint of phenomenal absence, i.e. as the essentially non-perceptible, abysmal centre of action that is the intellectual self. This kind of intersubjective relationship is the exact opposite of an objectifying assimilation by means of theoretical thought, for the universality of the intellect is of a completely different nature than the mere logical generality of created genera or species.^52^ 52 See Meister Eckhart, *Super Eccl.* no. 10, LW II, p. 239 (no English translation available). For the same reason, there can never be a subjective absorption of the Other through mystical fusion either. Being strictly incapable of receiving any *property* whatsoever, one intellectual *ego* can, by definition, never ap-*propriate* the other. This is the ultimate reason why Eckhart's thesis of the non-natural, uncreated character of the intellect does not equal metaphysical richness and power, but on the contrary, extreme poverty and nudity.

In Eckhart's approach, it is precisely thanks to its non-empirical mode of being that the fruitful, generative nature of intersubjective relationships can extend to all human beings alike, beyond the limits of empirical familial bonds. As much as our biological generation is responsible for the individual differences between human beings, as much our uncreated dimension of pure egoity unites us in the one eternal generation in which the Father engenders the Son, and in him, all intellectual beings.^53^ 53 See Meister Eckhart, *Pr*. 46, W I, pp. 490–491; for the English text, see *The Complete Mystical Works of Meister Eckhart*, pp. 131–132. To see the Other as *alter ego* means therefore to consider them from a perspective where we are for each other at the same time both “father” and “son”. The perfect reciprocity of this relationship can be literally interpreted as an-archy insofar as neither of the persons involved can dominate the other as their derivative product but only recognize them as co-original reflection of the generative origin in the absolute sense, i.e. the quelling life of the divine *ego*. This leads us to the conclusion that Eckhart's model of intersubjectivity, while still being governed by the paradigm of intellectual light and knowledge, implies nevertheless a radical renunciation to any pretence of power over, and empirically motivated “appropriation” of, the Other.

Thus, Eckhart's “egological” metaphysics anticipates in many ways Levinas’ ethical approach to subjectivity as being already essentially intersubjective, necessarily correlated to the Other and irreducible to any object-relation whatsoever. At the same time, however, Eckhart's thorough analysis of the particular linguistic role of *ego* as manifestation of an absolutely simple, accident-free substance appears as a blueprint for Derrida's defence of Husserl's conception of the Other as *alter ego* against Levinas’ criticism. What the French philosopher takes from the German Dominican is not so much the theological or, more precisely, Trinitarian background of his egological approach, but rather his subtle speculative grammar, which allows the encounter between *ego* and *alter ego* to be articulated according to a non-Aristotelian but nevertheless highly intelligible logic.

## 4. Derrida's “Third Way”

As is well known, Levinas’ cause of an “ethical overcoming” of traditional ontology and phenomenology has not gone unopposed. A substantial part of Derrida's essay “Violence and Metaphysics” is dedicated to a systematic questioning of the cornerstone assumptions in Levinas’ criticism of the apparent “ethical neutrality” in Husserl's and Heidegger's thought. Derrida's argumentative strategy is twofold: with regard to Heidegger, he highlights the fact that “letting be” (*Gelassenheit*) is not synonymous with indifference and anonymity, but refers to the primordial, non-ontic openness that constitutes the condition of the possibility to receive any kind of phenomena, including those which can never be transformed into an object.^54^ 54 See Derrida, “Violence and Metaphysics”, p. 138. Concerning Husserl's conception of the *alter ego*, Derrida seeks to prove that, despite appearances, this constitution of alterity does not amount to a tautological absorption of the Other into the self-same, but on the contrary, indicates true exteriority.^55^ 55 Ibid., pp. 122–123. In both cases, Derrida's argumentation appears visibly influenced by Eckhartian thought, although in a strangely complementary way. The explicit references to Eckhart's writings occur only in the part of his essay dealing with Heidegger, and focus mainly on the aspect of negative theology. His defence of Husserl's conception of alterity, by contrast, draws implicitly on one of the central aspects of Eckhart's transcendental egology without so much as mentioning the fact that for Eckhart, these two topics are intrinsically connected and therefore often expounded in the same sermons.

Derrida is well conscious of the Eckhartian roots of the motive of “letting be” in Heidegger and even quotes some of Eckhart's German sermons in the course of his essay, although with a slightly different philosophical intention. Whereas Heidegger considers Eckhart's *Gelassenheit* merely as an alternative to the Occidental definitions of subjectivity in terms of power and complete technical objectification of the world,^56^ 56 See Heidegger, *Discourse on Thinking*, pp. 58–90. Derrida is visibly more interested in Eckhart's idea of introducing primordial difference into God himself.^57^ 57 See Derrida, “Violence and Metaphysics”, p. 146. In one of his vernacular sermons, Eckhart stresses the fact that, just as every human being has to renounce their attachment to “this or that” particular created thing (*ens hoc et hoc*), God is not fit to encounter a *gelâzen* person in their utter simplicity unless He foregoes the particularity of the Trinitarian persons and accepts the poverty that reigns in the silent desert of the “deity”.^58^ 58 See Meister Eckhart, *Pr.* 2, *Werke* I, p. 35; for the English text, see *The Complete Mystical Works of Meister Eckhart*, p. 81. However, Derrida interprets the distinction between “God” (*got*) and “deity” (*gotheit*) as an indication that the German Dominican, despite his claims, still remains inside the logo-centric boundaries of negative theology. Instead of recognizing the “silent desert” as the divine equivalent to human *gelâzenheit* (i.e. as radical divestiture of any accidental property), Derrida considers this “silence” as the simple negation of traditional onto-theological discourse, i.e. not as potentially fertile, phenomenal openness, but as synonym for a hidden mystery that cannot be revealed to rational thought but only be reverently approached by humble faith.^59^ 59 See Almond, “How *Not* to Deconstruct A Dominican”, pp. 330–331.


Although Derrida's reading of Eckhart does admittedly more justice to the theological dimension of the German Dominican's thought than Heidegger's interpretation, he still fails to recognize the whole extent of his originality with regard to the mainstream of Occidental philosophical tradition. The crucial point is Eckhart's interpretation of the Trinity, which is precisely not identical with the God of onto-theology,^60^ 60 Concerning this point, the interpretation of Ian Almond does not seem to do fully justice to Eckhart, for although his Trinitarian God is always correlative to the created world, the creative action itself is of an essentially immanent, dynamic nature and therefore clearly distinguished from the transient, efficient causality that traditional onto-theology attributes to its *deus artifex* (see Almond, “Negative Theology, Derrida, and the Critique of Presence”, p. 152). but rather synonymous to quelling life itself, conceived as the primordial dynamic of thought. The nudity and nothingness that characterizes Eckhart's notion of the divine is not simply the result of the privative process of negative theology, but synonymous with the supra-categorical reality of the “I”, which is literally “no thing” to the precise extent that it is pure, energetic reality announcing itself as such. In “Violence and Metaphysics”, however, Derrida still interprets Eckhart's notion of God's “Being above Being” as an expression of ontic, quasi-spatial transcendence rather than in the metaphysical, Aristotelian sense, i.e. as the radical otherness and nothingness of the intellect with regard to its intelligible objects. Derrida writes:

And when Meister Eckhart seeks to go beyond these determinations, the movement which he sketches seems to remain enclosed in ontic transcendence. “When I said that God was not a Being and is above Being, I did not thereby contest his Being, but on the contrary attributed him a *more elevated Being*.” (*Quasi stella matutina … *). This negative theology is still a theology and, *in its literality at least*, it is concerned with liberating and acknowledging the ineffable transcendence of an infinite existent, “Being above Being and superessential negation”.^61^ 61 See Derrida, “Violence and Metaphysics”, p. 146 (emphasis is Derrida's).

Now, in the German sermon no. 9 (*Quasi stella matutina*) quoted by Derrida, Eckhart does indeed speak of attributing God a “Being higher than Being”, but the difference is not a merely quantitative or ontic one. In this vernacular text, Eckhart develops indeed the same radical difference between God's intellect and (created) being, which he had already expounded in his commentary on Exodus 3:14, the first Parisian Question, and other Latin texts. As Eckhart puts it in this sermon, being is merely the “forecourt”, i.e. an efflux and indirect manifestation of divine power, whereas reason alone is God's proper sanctuary and dwelling-place.^62^ 62 See Meister Eckhart, *Pr.* 9, W I, p. 109; for the English text, see *The Complete Mystical Works of Meister Eckhart*, p. 343. The sphere of the intellect is therefore not some subset of being but is above being in an absolute, supra-categorical sense.^63^ 63 See Meister Eckhart, *Pr.* 9, W I, p. 107; for the English text, see *The Complete Mystical Works of Meister Eckhart*, p. 342. In other words: the *puritas essendi* is to be understood as both a subjective and objective genitive. Intellect is the most pure form *of* being if we understand by “being” the plenitude of original, creative reality, and it is purified *from* being if we define “being” as the categorical mode of existence proper to created things. Either way, there is an ontological rift between the dynamic non-being (*unwesene*) of the intellect and the static, limited being (*wesene*) of this or that determinate thing.^64^ 64 See Meister Eckhart, *Pr.* 9, W I, p. 106; for the English text, see *The Complete Mystical Works of Meister Eckhart*, p. 342.


The “Being above Being” of the *Stella matutina* sermon is therefore nothing else but the accident-free, pure substance (*mera substantia*) that manifests itself in the groundless dynamic of the “I am who I am”. However, Derrida never actually accomplishes the passage from the negation of categorical ontology to the performative affirmation of hyper-categorical egoity, at least not in his explicit analysis of Eckhart's texts. His reading of Eckhart is ex-centric, so to speak, for he presents the German Dominican's thought as a more or less traditional configuration of negative theology without taking into account that Eckhart's relativization of classical ontology results from his particular theory of the intellect. Nevertheless, the true core of Eckhart's thought remains absently present, for Derrida's interpretation of Husserl's notion of *alter ego* refers exactly to the same form of transcendental grammar elaborated in Meister Eckhart's commentary on Exodus 3:14.

Derrida's analysis of Levinas’ charges against Husserl proceeds in two different steps, the first of which is still intra-phenomenological and the second, of Eckhartian inspiration. His strategy consists in contesting Levinas’ fundamental presupposition that Husserl's notion of intersubjectivity amounts to a tautological absorption of every form of alterity to the monolithic self-sameness of the *ego*. Derrida's begins his argumentation by putting forward the non-assimilatory character of intentionality as such. As Husserl has never failed to recall, even the phenomena of inner-worldly objects present themselves to the *ego* in a form of immanent transcendence that can never become a real part of the living stream of consciousness itself. In addition to this, spatial objects are never completely “given” in a saturated form of phenomenality, because their perception is necessarily marked by the limits of perspectivity and therefore always surrounded by a halo of indeterminacy and inactuality.^65^ 65 See Derrida, “Violence and Metaphysics”, pp. 118–126; Husserl, *Thing and Space*, pp. 95–97, 109. If this non-identity already holds good for inanimate objects, one can safely conclude that it must also be valid *a fortiori* with regard to the relationship between two different centres of consciousness which, by definition, can never become directly accessible to one another by means of immediate intuition. Husserl does not, however, analyze the grammatical implications of this radical non-continuity between *ego* and *alter ego*. He does speak about the “verbal sense” (*Wortsinn*) of the terms *alter* and *alter ego*,^66^ 66 See Husserl, *Cartesian Meditations*, pp. 110–111. but he never explicitly dwells on the syntactic particularity of this expression as opposed to the usual subject–predicate relation.

Unlike Husserl himself, Derrida does not limit himself to pointing out the radical exteriority of *ego* and *alter ego* on a pre-linguistic, phenomenal level, but proceeds to an analysis that shifts the grammar of egoity from the level of objectifying discourse to that of original self-manifestation. The following passage from “Violence and Metaphysics” is perhaps the one which sounds the most strikingly Eckhartian. Derrida writes:

Does not Levinas treat the expression *alter ego* as if *alter* were the epithet of a real subject (on a pre-eidetic level)? As an epithetical, accidental modification of my real (empirical) identity? Now, the transcendental syntax of the expression *alter ego* tolerates no relationship of substance to adjective, of absolute to epithet, in one sense or the other. This is its strangeness. [ … ] And this contradiction (in terms of a formal logic which Levinas follows for once, since he refuses to call the other *alter ego*), this impossibility of translating my relation to the Other into the rational coherence of language – this contradiction and this impossibility are not the signs of “irrationality”: they are the sign, rather, that one may no longer draw inspiration from *within* the coherence of the *Logos*, but that thought is stifled in the region of the origin of language as dialogue and difference.^67^ 67 Derrida, “Violence and Metaphysics”, pp. 127–128.

Derrida's phrase “the transcendental syntax of the expression *alter ego* tolerates no relationship of substance to adjective, of absolute to epithet, in one sense or the other” appears as an almost literal translation of what Eckhart says in his commentary on Exodus about the *ego* as pure substance that is not susceptible of any accidental predicate whatsoever.^68^ 68 “Li *ego* pronomen est primae personae. Discretivum pronomen meram substantiam significat; meram, inquam, sine omni accidente, sine omni alieno, substantiam sine qualitate, sine forma hac aut illa, sine hoc aut illo”. – “The *ego* is a first-person pronoun. As distinguishing pronoun it signifies the pure substance; the pure, I said, without any accident, without any foreign aspect, substance without quality, without this or that form, without this or that [aspect]” (Meister Eckhart, *In Exod*. no. 14, LW II, p. 20; the translation is ours). What Derrida intends to prove is that, despite appearances, the syntax of the *ego* can be interpreted neither according to the logic of ordinary language nor as its simple negation. Because the transcendental structure of the *ego* as living consciousness necessarily implies the continuous flow of immanent temporality, it does not form a stable substrate of predication, and what traditional grammar would call its determining adjective – the *alter* – denotes nothing but the distance with regard to another co-original but essentially discontinuous and inassimilable centre of consciousness. Derrida does therefore recognize the fundamental legitimacy of Levinas’ critique of traditional logic and ontology without, however, embracing the conclusion that the expression *alter ego* is still part of this paradigm of predication. The Greek conception of *logos* presents itself undoubtedly more often than not as a homogeneous sphere of thought capable of dealing with natural objects and human beings alike, according to the same categories. However, for Derrida, Husserl's conception of *alter ego* is no longer part of this logical continuum but, on the contrary, accomplishes the breakthrough toward “the origin of language as dialogue and difference”. The constitutive origin of language is something that cannot be spoken about in terms of constituted beings but has to be revealed according to its own transcendental language. This language of constitution does not denote static substrates and qualitative properties, but manifests centres of quelling generativity (*ego*) in their transcendental discontinuity and non-coincidence (*alter*). Curiously enough, though, Derrida presents his grammatical and syntactical analyses as if this idea was already present in Husserl himself, while in reality this approach is much closer to Eckhart's transcendental grammar of the “I” as absolute origin of intellectual self-manifestation.

Contrary to Levinas but in much the same way as Meister Eckhart, Derrida insists on the symmetric, mirror-like relationship between *ego* and *alter ego*. This does not mean that the Other is a mere reflection of my own stream of consciousness with all its individual intentional contents and memories, but that they are a reflection of the universal *form* of egoity we both have in common.^69^ 69 See Derrida, “Violence and Metaphysics”, p. 125. What the Other reflects me is the same phenomenal absence I experience with regard to the innermost source of my own life of consciousness, which is precisely not “my own” but withdrawn to a pre-egoic, pre-possessive level.^70^ 70 It is therefore somewhat inexact to affirm that Husserl fails to see the phenomenon of the unconscious and founds his phenomenology on the ideal of complete theoretical evidence and Cartesian self-awareness (see Large, “On the meaning of the word ‘other' in Levinas”, p. 48). Before “I” becomes “me”, there is the ungraspable breakthrough-point of transcendental temporalization I can never look towards but only away from.^71^ 71 See Husserl, *On the phenomenology of the Consciousness of Internal Time*, p. 79. The *alter ego* is therefore not an immanent spin-off of my own self, but on the contrary, the explicit external reminiscence and visible incarnation of what already “in” myself is irrevocably other than “me”.^72^ 72 “Dissymmetry itself would be impossible without this symmetry, which is not of this world, and which, having no real aspect, imposes no limit upon alterity and dissymmetry – makes them possible, on the contrary” (Derrida, “Violence and Metaphysics”, p. 126).


As Derrida points out, this relationship of reciprocal recognition is not conceived by Husserl as an inner-temporal process, where I am “first” enclosed in my own monadic *ego* and accidentally encounter “later on” someone whom I recognize as *alter ego*. In the §52 of the CM Derrida refers to, Husserl hints at the fact that the egoity of the Other is co-originally implied in the structure of my own monadic *ego*. “Inside” the monad, there is always already another monadic instance as something I have not “produced”, but which I recognize as a discontinuous manifestation of the same absolute, generative dynamic that constitutes my own stream of consciousness. Recognizing someone as *alter ego*, therefore, does not mean to constitute them as passive intentional object of our conscious activity of thinking, but to generate them “in” us (not in the sense of ontic inclusion but of reciprocal co-originality), *that is “in” themselves*, as equally groundless, playful centre of transcendental life that has always already engendered us as *its* Other.

Although Derrida's affinity to certain medieval thinkers may well have been prompted by the influence of his teacher Maurice de Gandillac,^73^ 73 Maurice de Gandillac translated, among others, texts of Meister Eckhart and Nicholas of Cusa. Derrida refers to his anthology of Cusanic texts in “Violence and Metaphysics”, p.101, fn. 34. there is also an intrinsic, systematic reason why Meister Eckhart in particular might have appeared to him as a potential ally in his critical debate with Levinas. Meister Eckhart's conception of the intellectual *ego* as being endowed with an essentially ec-static, dynamic correlation with other *egos* seems quite close to Levinas’ interpretation of ethical intersubjectivity as the most fundamental phenomenon of non-objective, inassimilable exteriority. Contrary to Levinas but much like Derrida,^74^ 74 See Wortham, “‘The discipline of the question’”, pp. 144–145. however, Eckhart puts particular emphasis on language as the privileged place where the non-worldly dignity of both *ego* and *alter ego* can manifest itself and be recognized. Against the intuitionist tendency in Levinas’ ethics, Eckhart insists on the fact that the divine nature of the Word in the absolute sense propagates itself like an infinite echo in the multiple words that compose human language.^75^ 75 “Words too have great power: we could work wonders with words. All words have their power from the first Word” (Meister Eckhart, *Pr.* 18, W I, p. 213; the English translation is taken from *The Complete Mystical Works of Meister Eckhart*, p. 213). Even if the intra-divine “difference” between the Father and the Son (which, by virtue of the radical phenomenal absence of its *relata*, might well be qualified as *différance* in Derrida's sense) is not directly accessible to intuitive experience, it announces itself through the event of difference inherent to language. Precisely because the primordial difference unfolds between the Father as absolute intellectual Origin and the Son as his only-begotten Word, there can be no true alterity and ethical recognition between human subjects without linguistic mediation either.

To be sure, Eckhart criticizes the inadequacy of Aristotelian categories and subject–predicate logic with regard to human subjectivity as unambiguously as Levinas. His answer, however, does not consist in replacing discursive language by awestruck, overwhelmed silence *vis-à-vis* the Other but in elaborating a different, more appropriate form of language, syntax, and transcendental grammar. In “Violence and Metaphysics”, Derrida follows exactly the same strategy, not only by emphasizing the essential role of language for any intersubjective experience, but also by stressing the fact that the expression *alter ego* already obeys a different transcendental grammar than Aristotelian predicative logic. The objectifying language that talks *about* people as if they were mere things gives way to a different linguistic paradigm, where the words are not simply used like prefabricated coins but generated and regenerated in their inalienable ec-static difference from to the intellect by the act of speaking.

For both Meister Eckhart and Derrida, the use of language is a participation in the original event of relationality as undefinable, accident-less difference between intellectual subjects. Eckhart, however, places the roots of this difference in God himself, more precisely: in His eternal *différance* with regard to his Word. While it is true that Derrida does not follow Eckhart in this openly theological interpretation of language, he nevertheless adopts Eckhart's egological foundation of a non-ontological, non-categorical grammar and syntax in the realm of human intersubjectivity. Despite this difference in detail, both the German Dominican and the French philosopher remain convinced that the specific medium of ethics and justice is not reverent silence in front of the Other, but rather an appropriate form of linguistic mediation and rational discourse.

## 5. Conclusion

In the light of our precedent analyses, we seem justified in concluding that Meister Eckhart's “egology of Exodus” provides an astonishing answer to the legitimate aspects of Levinas’ criticism of traditional Greek ontology without renouncing the ideal of theoretical knowledge as such. Eckhart's conception of the *ego* has emancipated itself from the paradigm of static substantiality by incorporating on a transcendental level the essential aspects of generative fertility and loving recognition Levinas had thought could only be found in the sphere of contingency and pre-philosophical everyday life. The important difference is, however, that Eckhart conceives the ethical encounter with the Other not as an immediate relationship between singular human beings, but as a relationship of relationships, where the first and primordial Other of each *ego* is not another human subject but the absolute Other of the divine *ego*. This distinction is important because it prevents us from putting excessive metaphysical strain on our fellow humans by making it their role to constitute our *ego* through the “call” of their alterity or by declaring ourselves their “hostages”.^76^ 76 See Levinas, *Otherwise than Being or Beyond Essence*, pp. 117–118. For Meister Eckhart, it is precisely because our *ego* is already constituted through its eternal, reciprocal generation from the absolute, divine *ego* that we can encounter other human beings without feeling threatened by their alterity but also without expecting of them what only God can give, i.e. our true self.

In “Violence and Metaphysics”, Derrida's proximity to Meister Eckhart is the most palpable in his considerations on the transcendental grammar of the expressions *ego*/*alter ego*, which clearly exceed Husserl's own argumentation frame in the CM. He also shares Eckhart's insight that the idea of a mirror-like relationship between *ego* and *alter ego* does not necessarily amount to a solipsistic or narcissistic conception of subjectivity, but on the contrary, expresses the transcendental openness of each “I” to its “Other” in an absolute sense. Like Levinas, Derrida links the specific ontological status of the subject to a certain form of nudity, but it is the transcendental nudity of the pure *ego*, which cannot hide its non-worldly strangeness behind some accidental property. The *ego* is, as it seems, exposed and vulnerable to the precise extent that its bareness is not merely factual and contingent, but transcendental and therefore never to be remedied by the ethical actions it inspires.

Eckhart's philosophical approach eludes the Levinasian distinction between the “Greek” and the “Jewish-biblical” paradigm not only because he draws equally on both lines of thought, but also for an intrinsic, systematic reason: with regard to rational subjectivity, these two adjectives are simply not applicable because they pertain to the empirical determinations of a person but have no bearing on the pure substance of their *ego* in its transcendental bareness. For Eckhart, neither *ego* nor *alter ego* are from this world (nor, *a fortiori*, from Athens or Jerusalem for that matter);^77^ 77 See Meister Eckhart, *In Ioh.* no. 450, LW III, p. 385 (no English translation available). rather, they open up and encompass the space of every possible world-constitution. The question of “identity” in its original sense has therefore nothing to do with our affiliation to certain empirical collectivities that define themselves in terms of determinate social, ethnic, cultural, or religious properties. Human identity as essentially *rational* identity is nothing but the original difference already at work in every egoic consciousness *as* the original, ex-centric orientation towards another intellectual *ego* equally devoid of any qualities whatsoever.

Derrida may have sensed this when towards the end of his essay he writes: “Are we Greeks? Are we Jews? But who, we?”^78^ 78 Derrida, “Violence and Metaphysics”, p. 153. Any attempt to determinate our who-ness by means of certain cultural, ethnic, or religious labels and paradigms is ultimately bound to fail in the light of the Eckhartian insight that not only with regard to God but with regard to every human being, the question “Who are you?” is actually no question at all, but already the response to the sheer self-manifestation of the “I am who I am”.

## References

[CIT0001] Aertsen Jan A. (1996). *Medieval Philosophy and the Transcendentals. The Case of Thomas Aquinas*.

[CIT0002] Almond Ian (1999). Doing Violence upon God: Nonviolent Alterities and Their Medieval Precedents. *Harvard Theological Review*.

[CIT0003] Almond Ian (2000). How *Not* to Deconstruct a Dominican: Derrida on God and ‘Hypertruth’. *Journal of the American Academy of Religion*.

[CIT0004] Almond Ian (1999). Negative Theology, Derrida, and the Critique of Presence: A Poststructuralist Reading of Meister Eckhart. *The Heythrop Journal*.

[CIT0006] Aristotle, *Metaphysics*.

[CIT0023] Bonaventure (1996). *On the Reduction of the Arts to Theology*.

[CIT0024] Bonaventure (1979). *Disputed Questions on the Mystery of the Trinity*.

[CIT0011] Colledge Edmund, McGinn Bernard (1981). *Meister Eckhart. The Essential Sermons, Commentaries, Treatises, and Defense*.

[CIT0012] Davies Oliver (2000). Beyond the Language of Being: A Comparative Study of Meister Eckhart and Emmanuel Levinas. *Eckhart Review*.

[CIT0013] Derrida Jacques (1987). Violence and Metaphysics. *Writing and Difference*, trans. Alan Bass.

[CIT0014] Eckhart Meister

[CIT0015] Eckhart Meister

[CIT0016] Eckhart Meister

[CIT0017] Eckhart Meister

[CIT0018] Eckhart Meister, Largier Niklaus (2008). *Werke*.

[CIT0019] Eckhart Meister

[CIT0020] Eckhart Meister

[CIT0021] Eckhart Meister

[CIT0022] Haworth Michael (2014). Telepathy and Intersubjectivity in Derrida, Husserl and Levinas. *Journal of the British Society for Phenomenology*.

[CIT0025] Heidegger Martin (1966). *Discourse on Thinking*.

[CIT0026] Heidegger Martin (1971). *On the Way to Language*.

[CIT0027] Heidegger Martin, Eugen Fink (1993). *Heraclitus*.

[CIT0028] Husserl Edmund (1999). *Cartesian Meditations. An Introduction to Phenomenology*.

[CIT0029] Husserl Edmund (1991). *On the Phenomenology of the Consciousness of Internal Time*.

[CIT0030] Husserl Edmund (1997). *Thing and Space*.

[CIT0031] Large William (1996). On the meaning of the word ‘other’ in Levinas. *Journal of the British Society for Phenomenology*.

[CIT0032] Levinas Emmanuel (1998). Is Ontology Fundamental?. *On Thinking-of-the-Other*.

[CIT0033] Levinas Emmanuel, (1990). *Difficult Freedom*.

[CIT0034] Levinas Emmanuel (1987). *Time and the Other*.

[CIT0035] Levinas Emmanuel (2002). *Totality and Infinity*.

[CIT0036] Levinas Emmanuel (1981). *Otherwise than Being or Beyond Essence*.

[CIT0037] Meir Ephraim (2009). Athens and Jerusalem in Levinas's *Difficult Freedom*. *Dialegesthai. Rivista telematica di filosofia*.

[CIT0038] Merleau-Ponty Maurice (1962). *Phenomenology of Perception*.

[CIT0039] Newheiser David (2012). Eckhart, Derrida, and the Gift of Love. *The Heythrop Journal*.

[CIT0040] Rigby Paul (2011). Levinas and Christian mysticism after Auschwitz. *Theological Studies*.

[CIT0041] Schwartz Yossef, Hasselhoff Görge K., Fraisse Otfried (2004). Meister Eckharts Schriftauslegung als maimonidisches Projekt. *Moses Maimonides (1138*–*1204). His Religious, Scientific, and Philosophical ‘Wirkungsgeschichte’ in Different Cultural Contexts*.

[CIT0042] Theologica, trans. The Fathers of the English Dominican Province (1947).

[CIT0043] van Riessen René D. N., Thomas Aquinas, Summa theologiae I, q. 27, a. 2 c. (2007). *Man as Place of God*.

[CIT0044] Walshe Maurice O'C. (2009). *The Complete Mystical Works of Meister Eckhart*.

[CIT0045] Wortham Simon Morgan (2015). The discipline of the question”: Rereading Derrida's “Violence and Metaphysics’. *Derrida Today*.

